# Toxoplasmosis presenting as a swelling in the axillary tail of the breast and a palpable axillary lymph node mimicking malignancy: a case report

**DOI:** 10.1186/1752-1947-5-348

**Published:** 2011-08-04

**Authors:** HP Priyantha Siriwardana, Louise Teare, Dia Kamel, E Reggie Inwang

**Affiliations:** 1 Department of Surgery, Broomfield Hospital, Court Road, Chelmsford, Essex, CM1 7ET, UK; 2 Department of Microbiology, Broomfield Hospital, Court Road, Chelmsford, Essex, CM1 7ET, UK; 3 Department of Pathology, Broomfield Hospital, Court Road, Chelmsford, Essex, CM1 7ET, UK

## Abstract

**Introduction:**

Lymphadenopathy is a common finding in toxoplasmosis. A breast mass due to toxoplasmosis is very rare, and only a few cases have been reported. We present a case of toxoplasmosis that presented as a swelling in the axillary tail of the breast with a palpable axillary lymph node which mimicked breast cancer.

**Case presentation:**

A 45-year-old otherwise healthy Caucasian woman presented with a lump on the lateral aspect of her left breast. Her mother had breast cancer that was diagnosed at the age of 66 years. During an examination, we discovered that our patient had a discrete, firm lump in the axillary tail of her left breast and an enlarged, palpable lymph node in her left axilla. Her right breast and axilla were normal. The clinical diagnosis was malignancy in the left breast. Ultrasound and mammographic examinations of her breast suggested a pathological process but were not conclusive. She had targeted fine-needle aspiration cytology (FNAC) and core biopsy of the lesions. FNAC was indeterminate (C3) but suggested a possibility of toxoplasmosis. The core biopsy was not suggestive of malignancy but showed granulomatous inflammation. She had a wide local excision of the breast lump and an axillary lymph node biopsy. Histopathology and immunohistochemical studies excluded carcinoma or lymphoma but suggested the possibility of intramammary and axillary toxoplasmic lymphadenopathy. The results of *Toxoplasma gondii *IgM and IgG serology tests were positive, supporting a diagnosis of toxoplasmosis.

**Conclusions:**

Toxoplasmosis rarely presents as a pseudotumor of the breast. FNAC and histology are valuable tools for a diagnosis of toxoplasmosis, and serology is an important adjunct for confirmation.

## Introduction

Lymphadenopathy is the most frequent clinical manifestation of acute infection with *Toxoplasma gondii *in the immunocompetent individual. Toxoplasma lymphadenitis typically involves a lymph node in the head and neck region, presents with or without systemic symptoms or extranodal disease, and runs a benign clinical course [1,2]. A breast mass due to toxoplasmosis is rare, and only a few cases have been reported [3-5]. We present a case of toxoplasmosis that presented as an axillary tail (breast) mass and a palpable axillary lymph node which mimicked breast cancer.

## Case presentation

A 45-year-old Caucasian woman with a left axillary tail (breast) mass and left-sided chest pain presented to the breast clinic. She also complained that her left breast had changed in appearance. She had a positive family history: her mother had breast cancer and her father had lung cancer. There was no nipple discharge, fever, or history of trauma to her breast. She had two children and had undergone a hysterectomy for benign disease two years before. Both of her ovaries were retained. There was no other significant medical history or known allergies. Her general health was good.

The result of a general examination was normal. There were two palpable nodules, one in the upper outer quadrant in the axillary tail of her left breast (20 mm) and the other in the left axilla (10 mm). The result of an examination of her right breast and axilla, abdomen, and other systems was normal. The most likely diagnosis was considered to be a malignant lesion in the left breast with metastatic involvement of an axillary lymph node.

She underwent ultrasound and mammographic examinations of her breasts. The mammogram showed a smooth-outlined, soft-density lesion in her left breast with no microcalcifications and a few small lymph nodes in her left axillary tail. Ultrasound revealed that the palpable lump in the lateral part of her left breast was a 2 cm solid lesion with reduced echogenicity. The other nodule, in the upper part of the left axilla, was also solid (1 cm) and suggestive of a lymph node (M4 U4; that is, suspicious abnormality according to the Breast Imaging Reporting and Data System, or BIRADS). The radiological appearance was highly suggestive of a lymphoma. Then she underwent targeted fine-needle aspiration cytology (FNAC) of the axillary lesion and core needle biopsy of the breast lesion. The FNAC was indeterminate (C3) but showed numerous monotonous lymphocytes in a background containing lymphogranular bodies suggestive of granulomatous inflammation such as toxoplasmosis. There were no malignant cells. The core biopsy showed a small aggregate of epitheleoid histiocytes and multinuclear giant cells in keeping with granulomatous inflammation. There was no evidence of a malignancy.

**Figure 1:**
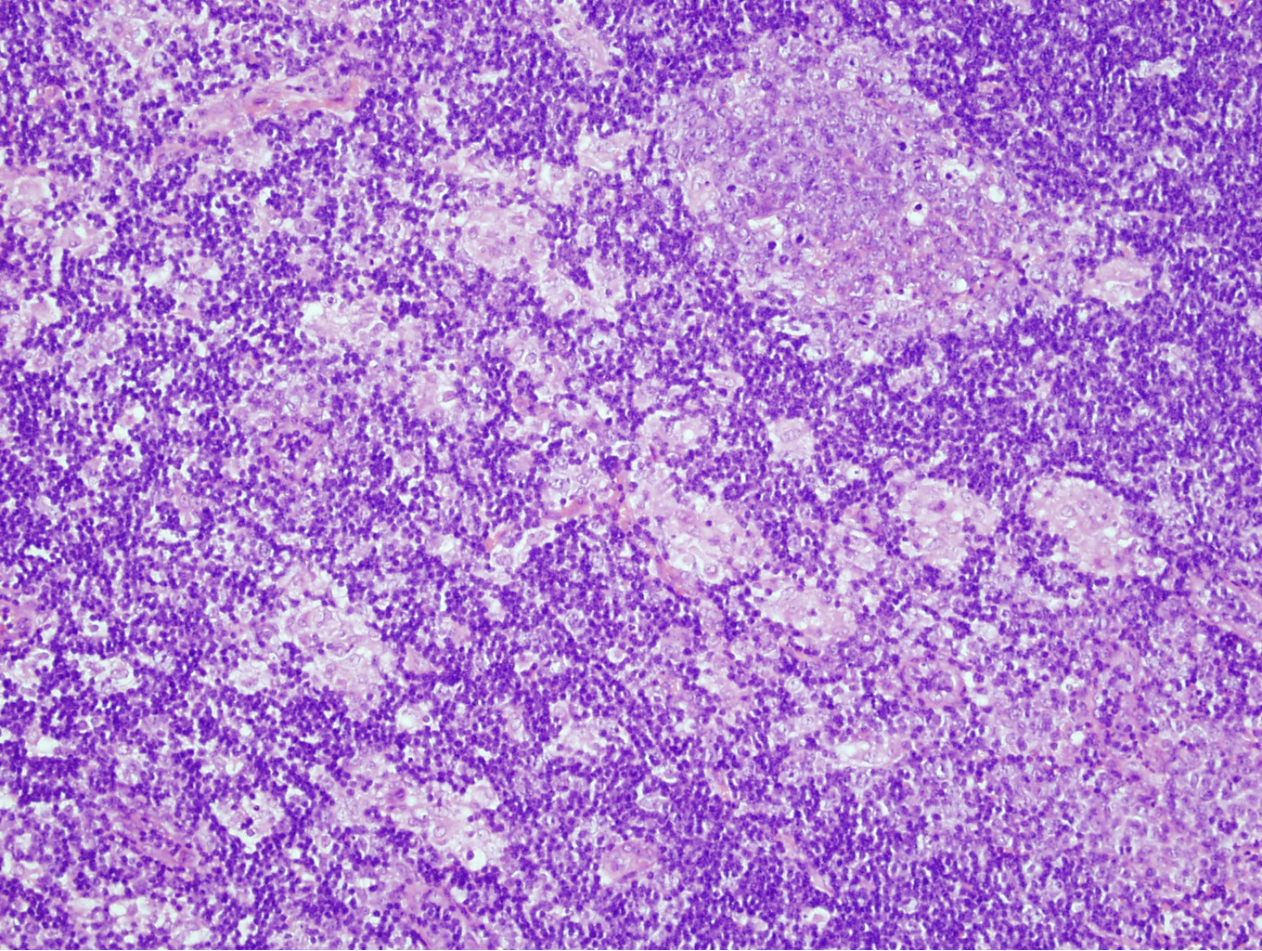
**A microscopic examination of the specimens of breast (axillary tail) lump and axillary lymph node shows marked follicular hyperplasia with prominent small granulomas composed almost entirely of epithelioid cells**.

**Figure 2:**
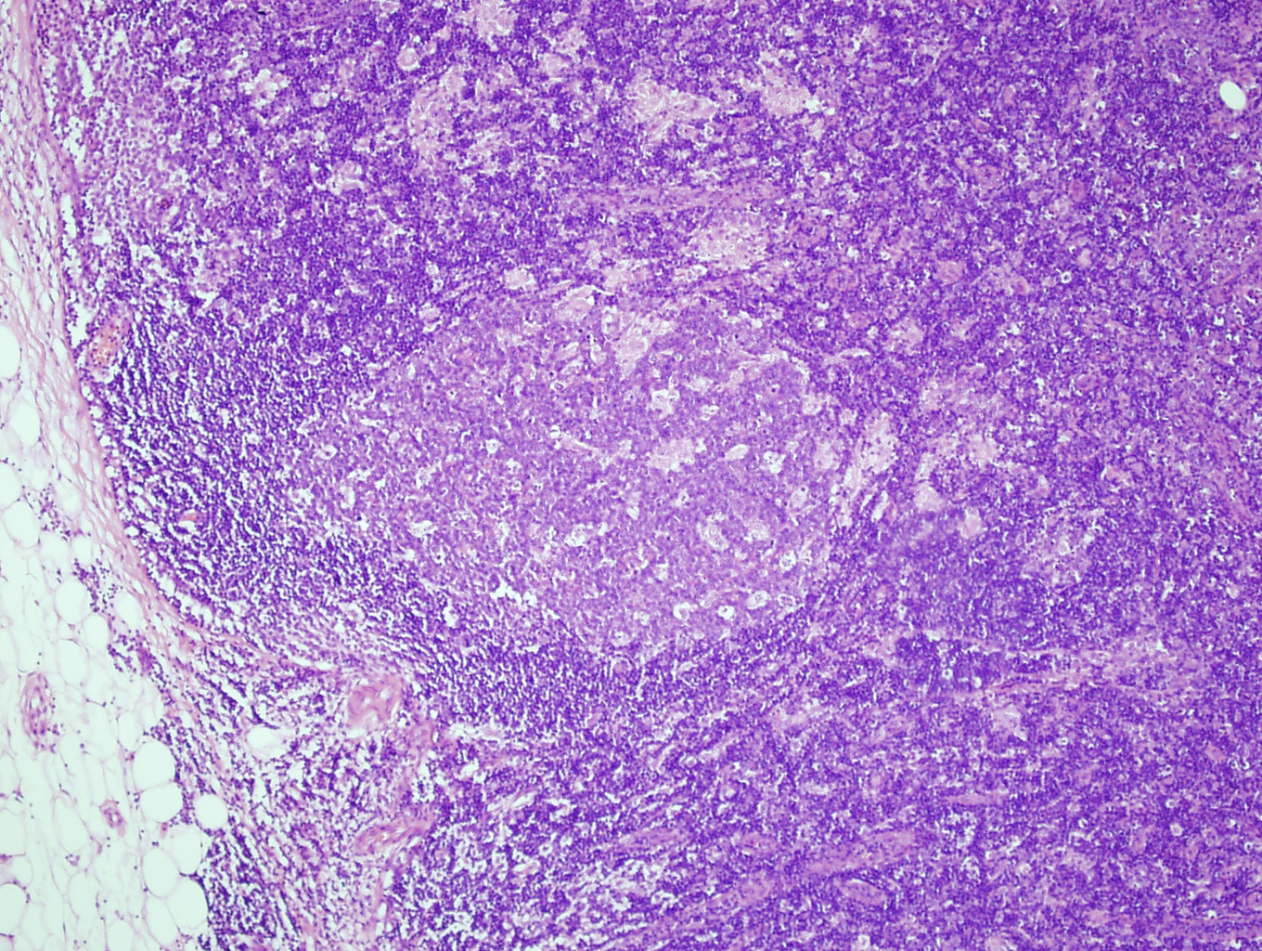
**A microscopic examination of the specimens of breast (axillary tail) lump and axillary lymph node shows marked follicular hyperplasia with prominent small granulomas composed almost entirely of epithelioid cells**.

Her case was discussed at the multidisciplinary meeting, and the team recommended a wide local excision of the breast lesion with palpable axillary lymph node biopsy. The results of a histological examination (Figures 1 and 2) of the resected specimens of breast and axillary lesions were suggestive of an intramammary and axillary lymph node mass with marked follicular hyperplasia. In addition, there were prominent microgranulomas composed almost entirely of epithelioid cells located within the hyperplastic follicles. Immunohistochemical staining showed an anatomical distribution of B- and T-cell markers. A Ziehl-Neelsen stain for acid-fast bacilli and Grocott and PAS+D (periodic acid-Schiff after diastase digestion) stains for fungi were negative. The histological appearances were similar to those described in toxoplasmosis, but the differential diagnoses included other infectious diseases and lymphadenopathy-associated autoimmune or immunodeficiency disorders. There were no features to suggest lymphoma or other malignancy. Histological material was referred for a second opinion that confirmed the above. The *T. gondii *serology tests detected Toxoplasma IgG and IgM antibodies suggestive of an acute or recently acquired Toxoplasma infection. Our patient was treated symptomatically as there were no indications to treat her toxoplamosis with antiprotozoal drugs. She has been well for the last two years since the diagnosis.

## Discussion

Toxoplasmosis is caused by infection with *T. gondii*, an obligate intracellular parasitic protozoa. The infection produces a wide range of clinical syndromes in humans, land and sea mammals, and various bird species. Toxoplasmosis passes from animals to humans, mainly via infected cat feces. *T. gondii *infects a large proportion of the world's population but rarely causes clinically significant disease. Although infection does not normally spread from person to person except through pregnancy, toxoplasmosis can, in rare instances, contaminate blood transfusions and organs donated for transplantation. In most immunocompetent individuals, primary or chronic (latent) *T. gondii *infection is asymptomatic in 80% to 90% of healthy hosts [1].

Lymphadenopathy is the most frequent manifestation of acute acquired infection in immunocompetent individuals. The typical presentation is a painless firm lymphadenopathy confined to one chain of nodes, most commonly cervical. Other physical manifestations include low-grade fever, hepatosplenomegaly, and skin rash. Our patient did not have any such manifestations.

Toxoplasma lymphadenitis most frequently involves a solitary lymph node in the head and neck region, presents with or without systemic symptoms or extranodal disease and runs a benign clinical course. However, serious extranodal disease does occur in a small percentage of patients and includes myocarditis, pneumonitis, encephalitis, chorioretinitis, and transmission of infection to the fetus [2]. Individuals at risk for severe or life-threatening toxoplasmosis include fetuses, newborns, and immunologically impaired patients. In immunodeficient individuals, toxoplasmosis most often occurs in those with defects of T cell-mediated immunity, such as those with hematologic malignancies, bone marrow and solid organ transplants, or AIDS.

Both histological features of biopsy specimens or cytology of needle aspirate [6] and serological tests are important in the diagnosis of toxoplasmosis and it was not until both were available in this case that a diagnosis of toxoplasmosis was made. The histological features have been well described [2] but sometimes can be confused with other disorders, particularly sarcoidosis, very early tuberculosis, cat-scratch disease [7], and more benign forms of Hodgkin disease, all of which may have a clinical presentation similar to that of toxoplasmosis [2]. Immunohistochemistry can help identify *T. gondii *within pathology specimens. Molecular polymerase chain reaction techniques have high specificity but low sensitivity in lymph node specimens, and the role of molecular biology in the diagnosis of toxoplasmosis has been reported [8]. Serology tests are an important adjunct but, on their own, must be interpreted with some care, as positive tests with low titers are common, presumably because of latent infection. In our case, however, serology testing was strongly positive, supporting the histological findings.

In an otherwise healthy person who is not pregnant, as in this case, treatment is not indicated. Symptoms will usually resolve within a few weeks [2]. If toxoplasmosis is acquired in pregnancy, transplacental infection may lead to severe disease in the fetus. Spiramycin may reduce the risk of transmission of maternal infection to the fetus. For people who have weakened immune systems, antiprotozoal drugs such as a combination of pyrimethamine and sulfadiazine are given for several weeks [2].

## Conclusions

Toxoplasmosis rarely presents as a mass in the axillary tail of the breast and may be considered as a differential diagnosis in patients presenting with axillary lymphadenopathy. FNAC and histology are valuable tools for a diagnosis of toxoplasmosis and serology is an important adjunct for confirmation. If the FNAC or core biopsy suggests the possibility of toxoplasmosis, serological investigations can confirm the diagnosis and may help avoid further invasive procedures and anxiety. Adult patients who are immunocompetent, are not pregnant and do not have involvement of a vital organ may be managed conservatively without antiprotozoal drugs.

## Abbreviation

FNAC: fine-needle aspiration cytology.

## Consent

Written informed consent was obtained from the patient for publication of this case report and any accompanying images. A copy of the written consent is available for review by the Editor-in-Chief of this journal.

## Competing interests

The authors declare that they have no competing interests.

## Authors' contributions

HPPS, the principal author, contributed to designing the report and writing the introduction, case presentation, and discussion sections. LT and DK contributed to the discussion. ERI collected the data, obtained consent from the patient, supervised the project, and undertook the final revision before submission. All authors read and approved the final manuscript.
